# Nitrogen isotope discrimination in open-pollinated and hybrid canola suggests indirect selection for enhanced ammonium utilization

**DOI:** 10.3389/fpls.2022.1024080

**Published:** 2022-11-11

**Authors:** Yi Hu, Robert D. Guy, Raju Y. Soolanayakanahally

**Affiliations:** ^1^ Key Laboratory of Mountain Surface Processes and Ecological Regulation, Institute of Mountain Hazards and Environment, Chinese Academy of Sciences, Chengdu, China; ^2^ Department of Forest and Conservation Sciences, Faculty of Forestry, University of British Columbia, Vancouver, BC, Canada; ^3^ Saskatoon Research and Development Centre, Agriculture and Agri-Food Canada, Saskatoon, SK, Canada

**Keywords:** nitrogen isotopes, carbon isotopes, nitrate, ammonium, model, canola

## Abstract

Nitrogen isotope discrimination (Δ^15^N) may have utility as an indicator of nitrogen use in plants. A simple Δ^15^N-based isotope mass balance (IMB) model has been proposed to provide estimates of efflux/influx (*E*/*I*) ratios across root plasma membranes, the proportion of inorganic nitrogen assimilation in roots (*P*_root_) and translocation of inorganic nitrogen to shoots (*Ti*/*Tt*) under steady-state conditions. We used the IMB model to investigate whether direct selection for yield in canola (*Brassica napus* L.) has resulted in indirect selection in traits related to nitrogen use. We selected 23 canola lines developed from 1942 to 2017, including open-pollinated (OP) lines developed prior to 2005 as well as more recent commercial hybrids (CH), and in three separate experiments grew them under hydroponic conditions in a greenhouse with either 0.5 mM ammonium, 0.5 mM nitrate, or 5 mM nitrate. Across all lines, *E*/*I*, *P_root_* and *Ti*/*Tt* averaged 0.09±0.03, 0.82±0.05 and 0.23±0.06 in the low nitrate experiment, and 0.31±0.06, 0.71±0.07 and 0.42±0.12 in the high nitrate experiment, respectively. In contrast, in the ammonium experiment average *E*/*I* was 0.40±0.05 while *Ti*/*Tt* averaged 0.07±0.04 and *P_root_* averaged 0.97±0.02. Although there were few consistent differences between OP and CH under nitrate nutrition, commercial hybrids were collectively better able to utilize ammonium as their sole nitrogen source, demonstrating significantly greater overall biomass and a lower *P_root_* and a higher *Ti*/*Tt*, suggesting a somewhat greater flux of ammonium to the shoot. Average root and whole-plant Δ^15^N were also slightly higher in CH lines, suggesting a small increase in *E*/*I*. An increased ability to tolerate and/or utilize ammonium in modern canola hybrids may have arisen under intensive mono-cropping.

## Introduction

Nitrogen (N) is the primary limiting nutrient for most plants growing in natural and agricultural ecosystems (Glass, 2003) and nitrate $\left(NO_{3}^{-}\right)$ and ammonium $\left(NH_{4}^{+}\right)$ are the two most important inorganic N sources for plants. Modern agricultural systems depend heavily on the use of N fertilizers for greater crop yield and more than 50% of the applied nitrogen is lost to the environment through leaching, greenhouse gas emissions (Vitousek et al., 1997; Hirel et al., 2007; Guo et al., 2010) and groundwater contamination (Guo et al., 2010). Canola is a relatively new crop, derived from oilseed rape (*Brassica napus* L.), and has become one of the world’s most important oil crops (Raymer, 2002). Like other non-legume crops, nitrogen is usually the limiting nutrient and N fertilizer is the biggest input needed for seed production (Wang et al., 2014; Zhang et al., 2022). Though very little is known about N uptake and assimilation in oilseed rape and canola, different N-use efficiencies among cultivars have been reported (Stahl et al., 2016; Li et al., 2020). Better understanding and characterization of whole-plant N-use and N-partitioning patterns among diverse canola lines is necessary for improving N-use efficiency (NUE), and beneficial in moving towards more sustainable agricultural production.

Both nitrate and ammonium have two major transport systems responsible for N uptake: high-affinity (HATS) and low-affinity (LATS) transport systems that are most effective at low or high N concentrations, respectively (Glass et al., 2002). Nitrate assimilation is more complex than ammonium. Once retained by the root, nitrate must first be reduced to nitrite and then ammonium before it can be assimilated further. Nitrate is converted into nitrite in the cytoplasm by nitrate reductase (NR), and nitrite is converted to ammonium by nitrite reductase (NiR). Ammonium, whether directly taken up by root cells or produced from nitrate, is assimilated into glutamine by glutamine synthetase (GS). It has been reported that under certain conditions, a substantial portion of the nitrate and/or ammonium initially taken up by plants (influx) returns to the rooting medium (efflux) before it can be assimilated (Kronzucker et al., 1997; Hawkins and Robbins, 2010). The ratio of efflux over influx (*E*/*I*) describes the bi-directional movement of inorganic N between root and rhizosphere and relates to the N-uptake efficiency; i.e., a low *E*/*I* indicates a high uptake efficiency because there is less leakage of inorganic N back to the rooting medium.

There are two stable isotopes of nitrogen (^14^N and ^15^N), with ^14^N being the predominant form (99.636% of global nitrogen). However, the stable isotopes of N may show differences in rates of chemical reaction, or in physical processes such as diffusion, that result in small changes in ^15^N/^14^N ratios between N pools. Changes in the relative abundance of ^15^N and ^14^N, called isotope fractionation, can provide integrated or tracer information about N fluxes within/through ecosystems (Evans, 2001; Robinson, 2001). It is well recognized that changes in plant δ^15^N occur during nitrate or ammonium assimilation, often causing plants to be depleted in ^15^N by about 2 to 3‰ compared to the soil N (Evans, 2001). This difference implies that there is discrimination against the heavier isotope, in either N transport or assimilation. It is now more than thirty years since the first reports of N isotope effects associated with NR and GS were measured *in vitro* using preparations from spinach leaves, yielding discrimination factors of ~15‰ for NR (Ledgard et al., 1985) and ~17‰ for GS (Yoneyama et al., 1993). Subsequent work has indicated that discrimination by either enzyme is probably somewhat higher (Needoba et al., 2004; Karsh et al., 2012; Carlisle et al., 2014; Cui et al., 2020). Nonetheless, under N-limited conditions, plant δ^15^N tends to be close to the δ^15^N value of the available soil inorganic N because local supplies of both isotopes are assimilated to near completion (Evans, 2001; Kolb and Evans, 2003). There is greater observed discrimination when substrate concentrations are high and/or there is a greater opportunity for residual heavy nitrogen to diffuse away from roots before being taken up again, but rarely (if ever) does the δ^15^N reflect the full discrimination factor of NR or GS (Yoneyama et al., 2001; Pritchard and Guy, 2005).

In a series of articles, Kalcsits and Guy (2013a), Kalcsits and Guy (2013b), Kalcsits and Guy (2014) presented an isotope mass balance (IMB) model that combines δ^15^N and tissue N content to derive (1) *E*/*I* at the root, (2) leaf *vs*. root partitioning of N assimilation, and (3) fluxes of inorganic and organic N to the shoot. Kalcsits and Guy (2013a), Kalcsits and Guy (2016a) and Hu and Guy (2020) took different approaches to assess the validity of the IMB model. The first approach was to test the IMB model by comparing it to a compartmental analysis of tracer efflux (CATE) using stable isotope tracing to determine root *E*/*I* in balsam poplar (*Populus balsamifera* L.). The highly correlated *E*/*I* from the two methods suggested the IMB model can be used for estimating N-uptake efficiency (1 – *E*/*I*), which is difficult to measure directly. In the second approach, the IMB model was tested by measuring the δ^15^N of inorganic and organic N forms in xylem sap in black cottonwood (*Populus trichocarpa* Torr. & Gray) to compare the calculated proportion of inorganic nitrogen assimilated in roots (*P_root_*) and translocation of inorganic nitrogen to shoots (*Ti*/*Tt*) to direct measurements. Hu and Guy (2020) validated and improved the model estimates by adjusting the discrimination factor for NR to 22‰. Significant genotypic variations in N-use traits from the IMB model were reported in balsam poplar under either $NO_{3}^{-}$ or $NH_{4}^{+}$ nutrition (Kalcsits and Guy, 2016b), and in black cottonwood (Hu and Guy, 2020) and heart-leaved willow (*Salix eriocephala* Michx.) under $NO_{3}^{-}$ (Hu et al., 2022).

For heart-leaved willow, Hu et al. (2022) used the IMB model and tissue C/N ratios to study variation in N-uptake efficiency and NUE, and stable carbon isotope analysis to study variation in water-use efficiency (WUE). The carbon isotopic composition (δ^13^C) of plant tissue can provide information on intrinsic water-use efficiency of C_3_ plants (Sun et al., 1996; Seibt et al., 2008). Fractionation of carbon isotopes occurs principally during diffusion of CO_2_ into the leaf and at fixation by RuBisCO, which both discriminate against ^13^C. Net discrimination can be simply modelled as a function of the atmosphere-to-leaf CO_2_ diffusion gradient, which also determines the intrinsic WUE of photosynthesis (Farquhar et al., 1982).

Canola has been under strong selection in breeding programs for higher yield and better oil quality. In this study, we selected 23 Canadian canola lines developed from 1942 to 2017, including open-pollinated (OP) lines developed prior to 2005 as well as more recent commercial hybrids (CH), and in three separate experiments grew them under hydroponic conditions in a greenhouse with either 0.5 mM ammonium, 0.5 mM nitrate or 5 mM nitrate. We hypothesized that direct selection for yield in canola may have resulted in the indirect selection of N-uptake efficiency, NUE and WUE.

## Material and methods

### Plant material, hydroponics system and experimental design

The experiment used 23 historical canola lines developed in Canada from 1942 to 2017, including open-pollinated lines developed prior to 2005 and more recent commercial hybrids (**Table 1**). Three separate experiments were conducted under hydroponic conditions in a greenhouse with either 0.5 mM ammonium, 0.5 mM nitrate (low nitrate), or 5 mM nitrate (high nitrate). Because of toxicity, it was not possible to grow plants in 5 mM ammonium.

**Table 1:** Historical *Brassica napus* L. lines sourced from Canadian canola breeding programs for use in the steady-state hydroponics study.

Year of release	Group	Seed source
1942	Open-pollinated	AAFC
1966	Open-pollinated	AAFC
1968	Open-pollinated	AAFC
1973	Open-pollinated	AAFC
1974	Open-pollinated	AAFC
1982	Open-pollinated	AAFC
1989	Open-pollinated	AAFC
1990	Open-pollinated	AAFC
1992	Open-pollinated	AAFC
1994	Open-pollinated	Raymond Gadoua
1996	Open-pollinated	AAFC
1998	Open-pollinated	University of Alberta
2001	Open-pollinated	Nutrien
2005	Open-pollinated	AAFC
2007	Commercial Hybrid	BASF
2011	Commercial Hybrid	Corteva
2012	Commercial Hybrid	Monsanto
2013	Commercial Hybrid	BASF
2014	Commercial Hybrid	Corteva
2014	Commercial Hybrid	Nutrien
2015	Commercial Hybrid	Corteva
2016	Commercial Hybrid	BASF
2017	Commercial Hybrid	Monsanto

AAFC (Agriculture and Agri-Food Canada); BASF (Baden Aniline and Soda Factory).

**Figure 1:**
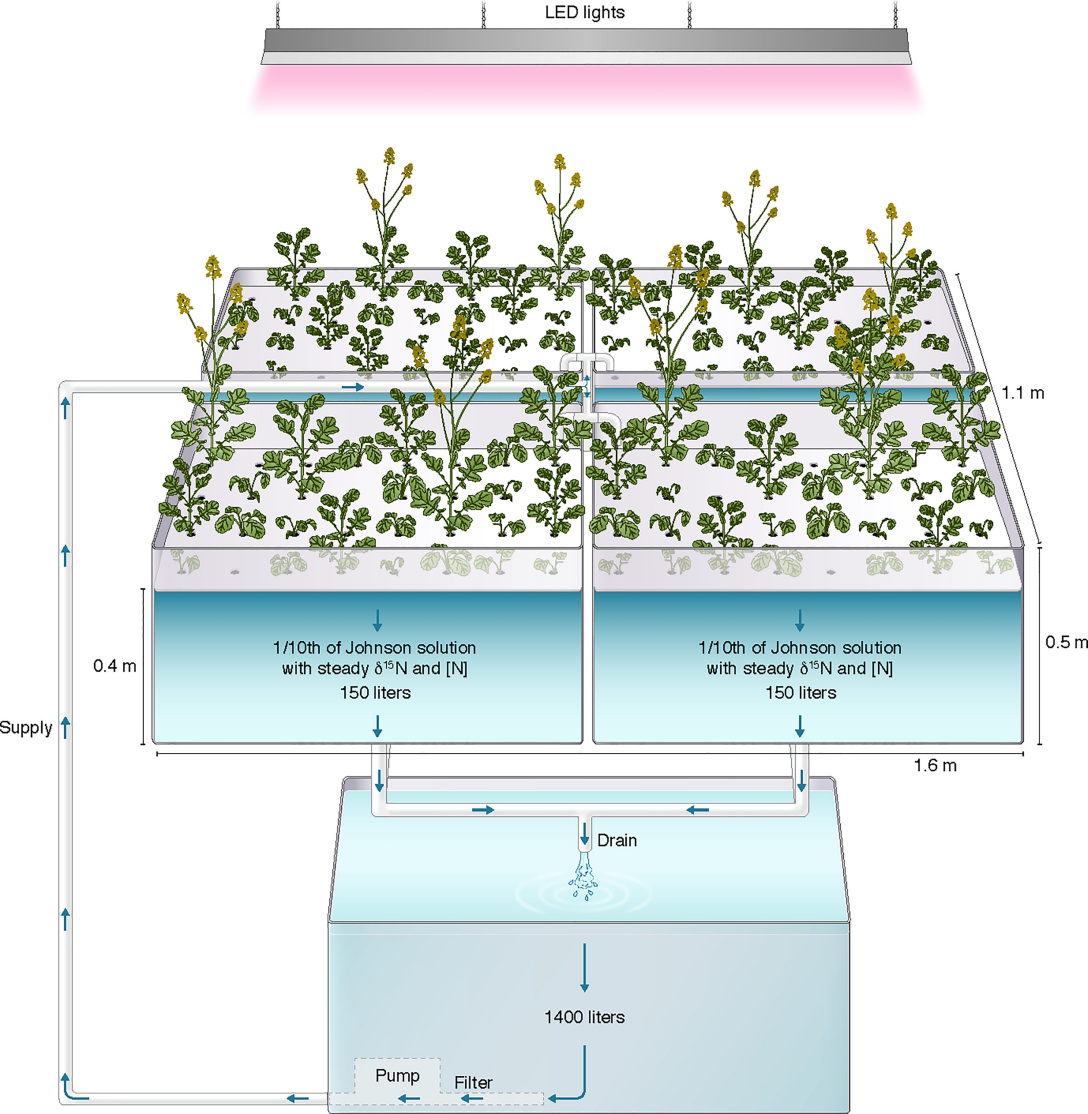
Simplified schematic of the greenhouse steady-state hydroponic system. Not shown are the aeration pump and a small receiving vessel containing a second submersible water pump returning media to the main reservoir. The artwork is drawn by Debbie Maizels, Zoobotanica - Science & Nature Illustration.

The hydroponic system (**Figure 1**) had four 150 L bench-mounted acrylic tubs, a 1400 L vertical ground tank (RK400; CANWEST, Surrey, Canada), two submersible water pumps (Little Giant, Fort Wayne, IN, USA) and connecting PVC piping (1-inch diameter; WaterTec, Langley, BC, Canada). One water pump sat in the bottom of the main tank and continuously circulated media to the four tubs, each holding a floating “raft” made of dense foam bolted between black (lower) and white (upper) sheets of Perspex. Each raft held 32 plants in drilled holes (2.5 cm, one plant per hole) fitted with partially slit foam plugs. For each experiment there were initially four replicate plants per line (one per raft, randomly arranged; extra holes were filled with additional canola plants that were not part of this study). The depth of water in the tubs was set by an overflow outlet draining into a receiving reservoir at ground level containing a float switch-activated pump that returned media to the main tank. The total volume of the system was maintained at 2000 L. To ensure aeration of the entire system, an air pump was connected to an air stone in the receiving reservoir. Exposed parts of the circulation system not including the rafts were covered with white/black (upper/inner) plastic to prevent algal growth. The system was under ambient light conditions supplemented by LED lighting (600 µmol m^-2^s^-1^) on an 18/6 h day/night photoperiod. Temperatures in the greenhouse were maintained between 20-24°C. To prevent overheating of the hydroponic solution, a 10 m cooling coil of ¼ inch stainless steel pipe was submersed in the main reservoir. Cold tap water flowed through the coil under the control of a thermostated solenoid valve (Red-Hat, Brantford, ON, Canada).

The hydroponics solution was a modified 1/10^th^ strength Johnson’s solution (Johnson et al., 1957) supplemented with either 0.5mM or 5mM nitrate (as Ca(NO_3_)_2_; δ^15^N= 3.5‰) for the low nitrate or the high nitrate experiment, respectively, and 0.5mM ammonium (as (NH_4_)_2_SO_4_; δ^15^N= 0.2‰) for the ammonium experiment. The solution was monitored daily for oxygen levels, pH and temperature. Powdered calcium carbonate (CaCO_3_) was added to buffer pH in the range of 6-7.5. Media $NO_{3}^{-}$ and $NH_{4}^{+}$ concentrations were measured periodically using the perchloric acid method (Cawse, 1967) and phenol:hypochlorite method (Solórzano, 1969). The solution was completely replaced every five days in an attempt to ensure there was no substantial decrease in $NO_{3}^{-}$ or $NH_{4}^{+}$ concentration over time that could result in major changes to the δ^15^N of the hydroponics solution. However, based on the total nitrogen content and isotopic composition of the harvested plants, 10.22, 21.26 and 2.36% of the supplied N was consumed in the ammonium, low nitrate and high nitrate experiments, which would have resulted in isotopic enrichments of 0.5, 0.4 and 0.1‰, respectively. Accordingly, source δ^15^N values were adjusted by these amounts for purposes of calculating discrimination.

### Isotope analysis and IMB model calculations

Canola plants were harvested into leaves, stems, and roots after 45 days of growth. Once freeze-dried, these parts were weighed and then pulverized using a Wiley mill (Fritsch Laborgeratebau, Terochem Scientific, Ottawa, ON, Canada) followed by a Geno/Grinder (SPEX SamplePrep, Metuchen, New Jersey, USA). Sub-samples (3 mg) were packed into tin capsules and analyzed for %C, %N, δ^13^C and δ^15^N using a Vario EL Cube Elemental Analyzer (Elementar, Germany) interfaced to an Isoprime Isotope Ratio Mass Spectrometer (GV Instruments, UK) in the Stable Isotope Lab, Agriculture and Agri-Food Canada Lethbridge Research and Development Centre, Lethbridge, Alberta, Canada.

**Figure 2:**
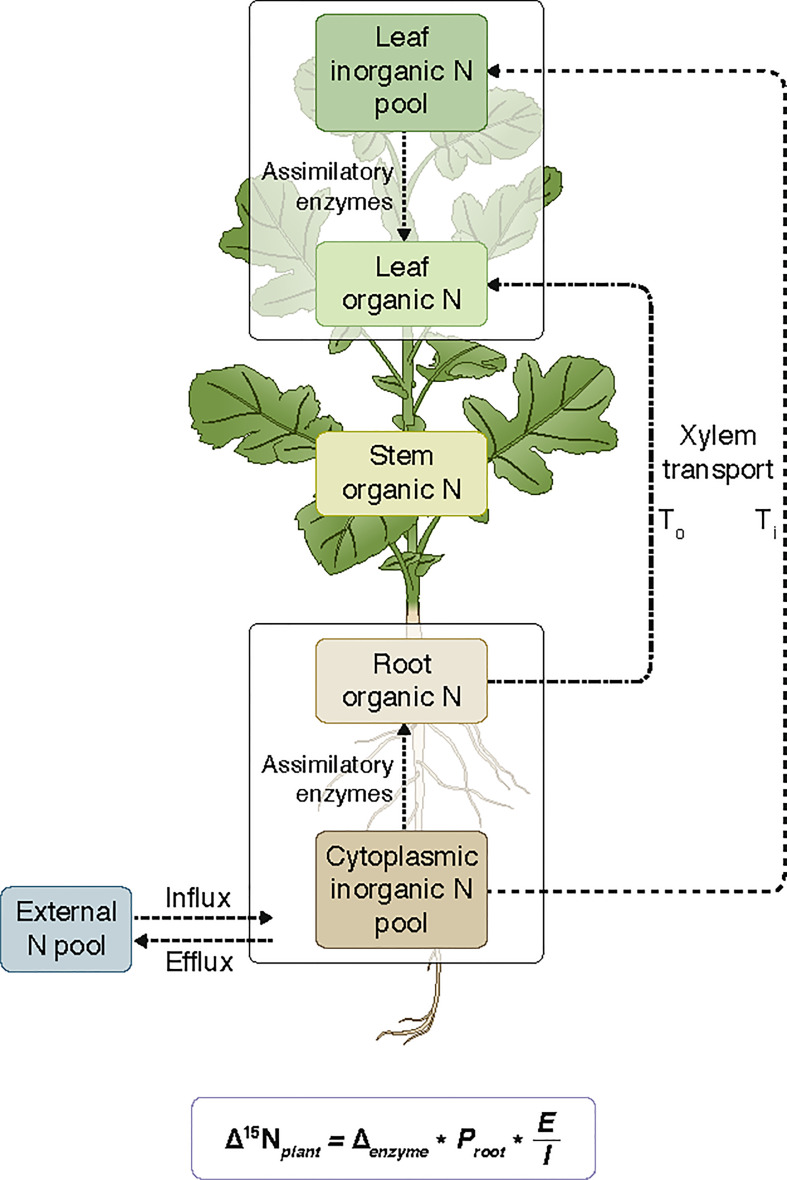
Diagram of *Brassica napus* L. nitrogen fluxes during the uptake of inorganic nitrogen from the external pool and assimilation into either root or leaf organic nitrogen as proposed in the isotope mass balance model. Arrows refer to unidirectional fluxes of nitrogen between leaf and root pools. *T*_o_ represents the organic nitrogen translocated to the leaves and *T*_i_ represents the inorganic nitrogen translocated to the leaves. Stem tissue is assumed to arise from a mixture of leaf and root organic pools, after Kalcsits et al. (2014). The artwork is drawn by Debbie Maizels, Zoobotanica - Science & Nature Illustration.


Nitrogen isotopic composition (δ^15^N) and isotope discrimination (Δ) were expressed as:


(1) $$\delta^{15}N=\left(\frac{R_{sample}}{R_{standard}}-1\right)*1000$$

(2) $$\Delta^{15}N_{sample}=\frac{\left(\delta^{15}N_{source}-\delta^{15}N_{sample}\right)}{\left(1+\delta^{15}N_{sample}/1000\right)}$$

where, *R*_sample_ and *R*_standard_ are the ^15^N/^14^N ratios of the sample and the arbitrary standard (air N_2_), respectively. Plants were divided into three major parts (leaf, stem, and root), and the whole-plant isotope discrimination was calculated as the weighted sum of these parts:


(3) $$\Delta^{15}N_{whole-plant}=(f_{root}\times \Delta^{15}N_{root})+(f_{stem}\times \Delta^{15}N_{stem})+(f_{leaf}\times \Delta^{15}N_{leaf})$$

where, Δ^15^N*_i_* is the discrimination relative to the external media N source and *f_i_* is equal to the fraction of tissue nitrogen contributing to overall plant nitrogen content. The percent ratio of inorganic nitrogen (*Ti*) relative to total nitrogen (*Tt*) translocated to the leaves (*Ti*/*Tt*), as predicted by the IMB model (Kalcsits et al., 2014, **Figure 2**), was calculated as:

(4) $$\frac{Ti}{Tt}=\frac{\left(\Delta^{15}N_{root}-\Delta^{15}N_{leaf}\right)}{\Delta_{enzyme}}*100$$

where, Δ*_enzyme_* is the discrimination factor for NR (22‰) or GS (17‰), as appropriate for either nitrate or ammonium assimilation. Assuming leaves and roots are the major sites of nitrogen assimilation, the amount of stem N (relative to total plant N) that originated from the leaves (*f_stem-leaf_*) was approximated by:


(5) $$f_{stem-leaf}=\frac{\Delta^{15}N_{stem}-\Delta^{15}N_{root}}{\Delta^{15}N_{leaf}-\Delta^{15}N_{root}}\times f_{stem}$$

and the proportion of total plant nitrogen assigned to the leaf pool was:


(6) $$f_{leaf\;pool}=f_{leaf}+f_{stem-leaf}$$

Δ^15^N*_stem_* frequently exceeded Δ^15^N*_root_* in the ammonium experiment, in which case all stem N was assumed to originate from the roots and *f_stem–leaf_* was assigned a value of zero. The proportion of total plant nitrogen assimilated in the roots (*P_root_*) was given by:


(7) $$P_{root}=1-\left(f_{leaf\;pool}\times \frac{Ti}{Tt}\right)$$

and efflux over influx (*E*/*I*) was then calculated as:


(8) $$\frac{E}{I}=\frac{\Delta^{15}N_{plant}}{\Delta_{enzyme}\times P_{root}}$$

### Statistics

All statistical analyses used R version 3.5.1 (R Core Development Team, 2022). One-way nested ANOVA was used to test for differences in plant biomass, whole-plant and tissue C and N concentrations, whole-plant and tissue δ^13^C and δ^15^N, and IMB model estimates with group effect (OPs and CHs) as a fixed factor and historical lines nested within the group effect. Pearson correlation coefficients (*r*) were used to examine relationships between all physiological variables determined among the 23 canola lines.

## Results

Plants reached greater size in the nitrate experiments than in the ammonium experiment, but given that these were all separate experiments, we are unable to test for differences in performance between N sources or concentrations. There were no obvious signs of N limitation or toxicity in all three experiments. Foliar nitrogen averaged (±SD) 4.48±0.69% in the 0.5mM (low) nitrate experiment, 4.90±0.94% in the 5 mM (high) nitrate experiment, and 5.64±1.27% in the 0.5 mM ammonium experiment, which is within the expected 3-6% sufficiency range for herbaceous plants (Römheld, 2012).

Plants showed considerable discrimination against ^15^N in the ammonium experiment. Across all 23 canola lines, the leaf, stem and root Δ^15^N averaged (±SD) 6.1±0.8‰, 8.2±0.9‰ and 7.1±1.0‰, respectively (*P*< 0.001) Group means, ranges and statistical differences are summarized in **Table 2**. There were clear statistical differences between OP and CH lines for whole-plant biomass, root Δ^15^N, whole-plant Δ^15^N and whole-plant δ^13^C. The difference in the whole-plant Δ^15^N can largely be ascribed to the difference in root Δ^15^N. The CH group had 76% higher biomass than the OP group, while the root:shoot ratios were similar. The CH group also had significantly higher average root and whole-plant Δ^15^N than the OP group, by 0.6‰ and 0.3‰, respectively. For whole-plant δ^13^C, the CH group was 0.5‰ higher than the OP group. Although the differences were small, the IMB model estimates suggested significantly higher *E*/*I* and *Ti*/*Tt*, and lower *P*_root_ in the CH group as compared to the OP group. There was no statistical difference between OP and CH lines in whole-plant C/N, being 9.95±2.03 for the OP group and 10.40±2.44 for the CH group, respectively.

**Table 2:** Overall means and ranges of whole-plant (WP) biomass, root-to-shoot (R:S) ratio, organ and whole-plant level nitrogen isotope discrimination (Δ^15^N) and related isotope mass balance (IMB) model estimates, whole-plant carbon isotope composition (δ^13^C) and carbon to nitrogen ratio (C/N) between open-pollinated (OP) lines and commercial hybrids (CH) in *Brassica napus* L. grown under 0.5 mM ammonium.

Trait	Open-pollinated (OP) lines			Commercial hybrids (CH)			Group difference
		Mean value ± SD	Data range	Mean value ± SD	Data range		
WP biomass	(g)	3.25 ± 1.24	0.68–5.68	5.70 ± 2.15	1.15–9.98	*P*<0.001	
R:S ratio		0.24 ± 0.07	0.06–0.38	0.25 ± 0.08	0.05–0.39	*n.s.*	
Leaf Δ^15^N	(‰)	6.0 ± 0.8	3.8–7.7	6.1 ± 0.9	3.9–7.7	*n.s.*	
Root Δ^15^N	(‰)	6.9 ± 1.0	3.8–9.2	7.5 ± 0.8	4.7–8.9	*P*<0.001	
Stem Δ^15^N	(‰)	8.1 ± 1.0	5.2–9.8	8.2 ± 0.9	5.6–9.8	*n.s.*	
WP Δ^15^N	(‰)	6.5 ± 0.8	4.0–8.3	6.8 ± 0.8	5.1–7.9	*P*<0.05	
*Ti*/*Tt*		0.05 ± 0.04	0.01–0.15	0.08 ± 0.04	0.01-0.16	*P*<0.001	
*P_root_*		0.98 ± 0.02	0.88–0.99	0.96 ± 0.03	0.88–0.99	*P*<0.001	
*E*/*I*		0.39 ± 0.05	0.23–0.51	0.41 ± 0.05	0.29–0.50	*P*<0.01	
WP δ^13^C	(‰)	-31.6 ± 1.0	-33.5– -29.3	-31.1 ± 1.0	-33.0– -29.5	*P*<0.05	
WP C/N		9.95 ± 2.03	6.28–17.21	10.4 ± 2.44	6.88–16.63	*n.s.*	

n.s., not significant.

Unlike the ammonium experiment, there were no statistically significant differences between the OP and the CH groups in either the low nitrate experiment (**Table 3**) or the high nitrate experiment (**Table 4**). In the low nitrate experiment, the average whole-plant biomass across all lines was 9.55±7.65 g and the average root:shoot ratio was 0.14±0.05. Mean leaf, stem and root Δ^15^N was 0.7±0.8‰, 1.7±0.8‰ and 5.7±1.3‰, whereas whole-plant Δ^15^N and δ^13^C were 1.7±0.6‰ and -30.8±0.7‰, respectively. Estimated *E*/*I*, *P_root_* and *Ti*/*Tt* averaged 0.09±0.03, 0.82±0.05 and 0.23±0.06, respectively.

**Table 3:** Overall means and ranges of whole-plant (WP) biomass, root-to-shoot (R:S) ratio, organ and whole-plant level nitrogen isotope discrimination (Δ^15^N) and related isotope mass balance (IMB) model estimates, whole-plant carbon isotope composition (δ^13^C) and carbon to nitrogen ratio (C/N) between open-pollinated (OP) lines and commercial hybrids (CH) in *Brassica napus* L. grown under 0.5 mM nitrate condition.

Trait	Open-pollinated (OP) lines			Commercial hybrids(CH)		Group difference
		Mean value ±SD	Data range	Mean value ±SD	Data range	
WP biomass	(g)	9.62 ± 8.24	0.75–33.60	9.39 ± 7.13	0.96–28.01	*n.s.*
R:S ratio		0.14 ± 0.04	0.04–0.23	0.13 ± 0.05	0.04–0.24	*n.s.*
Leaf Δ^15^N	(‰)	0.7 ± 0.9	-0.7–2.5	0.4 ± 0.7	-1.7–1.9	*n.s.*
Root Δ^15^N	(‰)	5.7 ± 1.0	2.9–7.8	5.6 ± 1.5	1.9–8.4	*n.s.*
Stem Δ^15^N	(‰)	1.8 ± 0.7	0.6–3.7	1.5 ± 1.0	-0.2–3.6	*n.s.*
WP Δ^15^N	(‰)	1.7 ± 0.5	0.9–3.1	1.5 ± 0.6	0.5–3.2	*n.s.*
*Ti*/*Tt*		0.23 ± 0.06	0.10–0.38	0.24 ± 0.07	0.11-0.35	*n.s.*
*P_root_*		0.82 ± 0.05	0.70–0.94	0.81 ± 0.07	0.77–0.96	*n.s.*
*E*/*I*		0.09 ± 0.03	0.05–0.17	0.08 ± 0.03	0.03–0.16	*n.s.*
WP δ^13^C	(‰)	-30.8 ± 0.7	-32.4– -29.0	-31.0 ± 0.7	-32.9– -29.8	*n.s.*
WP C/N		12.64 ± 2.01	8.35–17.82	12.52 ± 2.25	9.99–17.93	*n.s.*

n.s., not significant.

**Table 4:** Overall means and ranges of whole-plant (WP) biomass, root-to-shoot (R:S) ratio, organ and whole-plant level nitrogen isotope discrimination (Δ^15^N) and related isotope mass balance (IMB) model estimates, whole-plant carbon isotope composition (δ^13^C) and carbon to nitrogen ratio (C/N) between open-pollinated (OP) lines and commercial hybrids (CH) in *Brassica napus* L. grown under 5 mM nitrate condition.

Trait	Open-pollinated (OP) lines			Commercial hybrids(CH)		Group difference
		Mean value ± SD	Data range	Mean value ± SD	Data range	
WP biomass	(g)	11.62 ± 6.31	1.88–24.06	12.41 ± 6.81	0.89–26.3	*n.s.*
R:S ratio		0.16 ± 0.15	0.02–0.96	0.19 ± 0.12	0.04–0.48	*n.s.*
Leaf Δ^15^N	(‰)	2.0 ± 2.5	-3.5–6.4	1.7 ± 2.3	-2.3–5.7	*n.s.*
Root Δ^15^N	(‰)	11.2 ± 0.9	8.5–12.2	11.3 ± 0.8	8.8–12.4	*n.s.*
Stem Δ^15^N	(‰)	6.0 ± 1.1	3.4–7.8	6.1 ± 1.5	3.5–9.1	*n.s.*
WP Δ^15^N	(‰)	4.7 ± 1.4	1.2–7.1	4.8 ± 1.3	3.0–8.0	*n.s.*
*Ti*/*Tt*		0.42 ± 0.12	0.15–0.71	0.43 ± 0.11	0.28-0.63	*n.s.*
*P_root_*		0.71 ± 0.07	0.55–0.82	0.71 ± 0.05	0.62–0.85	*n.s.*
*E*/*I*		0.30 ± 0.07	0.08–0.40	0.31 ± 0.06	0.20–0.43	*n.s.*
WP δ^13^C	(‰)	-31.5 ± 0.8	-33.7– -30.2	-31.5 ± 0.6	-32.5– -30.3	*n.s.*
WP C/N		8.04 ± 1.56	5.06–11.00	8.36 ± 1.76	4.59–12.63	*n.s.*

n.s., not significant.

In the high nitrate experiment, whole-plant biomass averaged 11.92±6.65 g and the mean root:shoot ratio was 0.16±0.13. Mean values for the leaf, stem and root Δ^15^N were 2.0±2.4‰, 6.0±1.2‰ and 11.3±0.9‰, and the whole-plant Δ^15^N and δ^13^C were 4.8±1.3‰ and -31.5±0.6‰, respectively. The average *E*/*I* was 0.31±0.06, while *P_root_* was 0.71±0.07 and *Ti*/*Tt* was 0.42±0.12.

**Figure 3:**
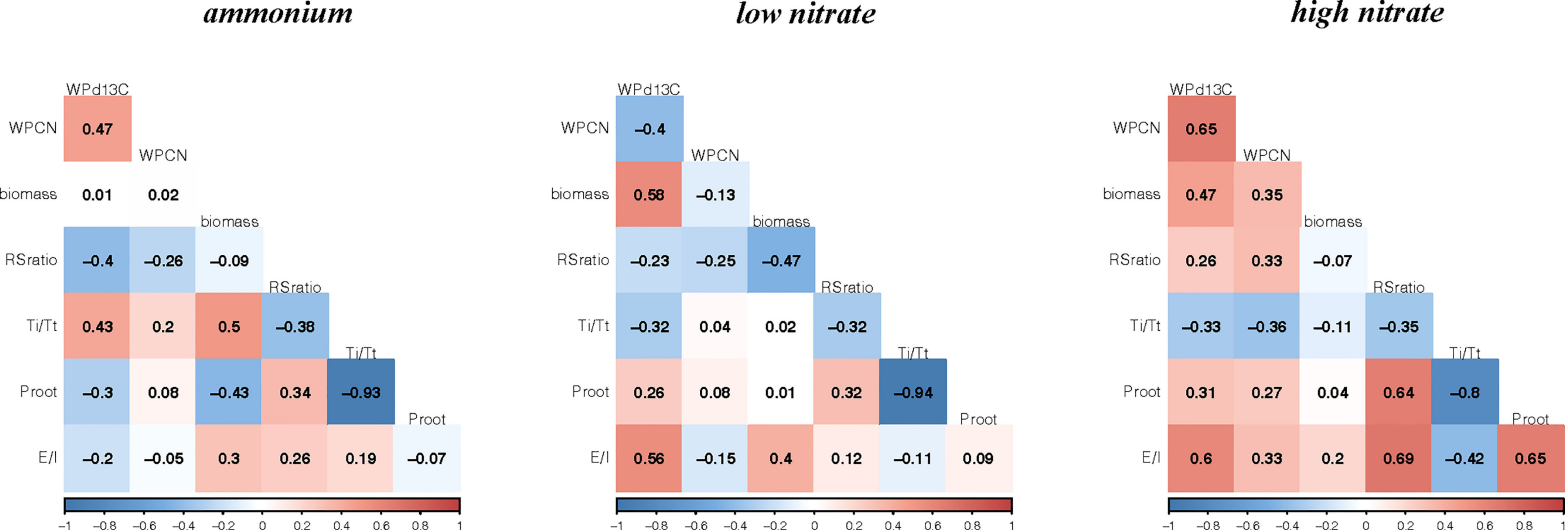
Heat-map of genetic correlations (r) for mean biomass and physiological traits for 23 historical canola lines grown with ammonium (0.5 mM), low nitrate (0.5 mM) or high nitrate (5 mM). WP, whole-plant; C/N, carbon to nitrogen ratio; Biomass, g; δ^13^C, carbon isotope composition; *Ti*/*Tt*, the proportion of inorganic nitrogen transported from roots to leaves; *P_root_*, proportion of inorganic nitrogen assimilated in the roots; *E*/*I*, root efflux/influx ratio.

Genetic correlations between growth and isotope-based physiological traits in each experiment are shown in **Figure 3**. *P_root_* and *Ti*/*Tt* were consistently and very strongly negatively related to each other in all three experiments (*P*< 0.001), as would be expected based on Equation 7. Otherwise, the strength and direction of trait-to-trait correlations varied considerably between experiments.

In the ammonium experiment, whole-plant biomass was positively correlated to *Ti*/*Tt* (*P*< 0.05), and negatively to *P_root_* (*P*< 0.05). Root:shoot ratio was almost but not quite significantly negatively correlated to whole-plant δ^13^C (*P* = 0.058). *P_root_* was negatively correlated with biomass (*P*< 0.05). Whole-plant δ^13^C was significantly and positively correlated with the whole-plant C/N ratio (*P*< 0.05).

In the low nitrate experiment, biomass was positively correlated to whole-plant δ^13^C (*P*< 0.01) but not quite *E*/*I* (*P* = 0.061), and negatively correlated to root:shoot ratio (*P*< 0.05). Similar to the ammonium experiment, *E*/*I* was not correlated with either *Ti*/*Tt* or *P_root_*, however *P_root_* was also not significantly correlated with biomass. In sharp contrast to the ammonium experiment, whole-plant δ^13^C was positively related to *E*/*I* (*P*< 0.01) and negatively related to whole-plant C/N, but not quite significantly so (*P* = 0.063).

In the high nitrate experiment, biomass was positively correlated only to whole-plant δ^13^C (*P*< 0.05). In contrast to both the ammonium and the low nitrate experiments, root:shoot ratio was significantly positively correlated to *P_root_* and *E*/*I* (*P*< 0.001). Further, *P_root_* was significantly and strongly correlated to *E*/*I* (*P*< 0.001). In concordance with the ammonium experiment, but not the low nitrate experiment, whole-plant δ^13^C was positively related to whole-plant C/N (*P*< 0.001). By contrast, in concordance with the low nitrate experiment, but not the ammonium experiment, whole-plant δ^13^C was positively related to *E*/*I* (*P*< 0.001).

## Discussion

There are three major competing fates for inorganic N taken up by roots, including (1) assimilation in the root cytosol, (2) loading of unassimilated N into the xylem for root-to-shoot translocation, or (3) efflux back to the rooting medium (Glass, 2003). Since the assimilating enzymes (NR and GS) prefer lighter N (^14^N) over heavier N (^15^N) during assimilation, organic N in plants is generally depleted in ^14^N while remaining unassimilated inorganic N is enriched (Ledgard et al., 1985; Yoneyama et al., 1993). In this study, we found that root and whole-plant Δ^15^N were both positive across all three experiments, indicating that lighter inorganic N forms ${(}^{14}NH_{4}^{+}or{\;}^{14}NO_{3}^{-})$ were preferentially assimilated by plants while some fraction of the heavier unassimilated inorganic N ${(}^{15}NH_{4}^{+}\;or{\;}^{15}NO_{3}^{-})$ returned to the hydroponic medium. Although the three experiments were not conducted simultaneously, mean whole-plant Δ^15^N was highest in the ammonium experiment (6.6‰) and lowest in the low nitrate experiment (1.7‰), with the high nitrate experiment in the middle (4.8‰). The leaf Δ^15^N, which represented up to 70% of plant N in this study and is often used as a proxy for whole-plant Δ^15^N, also followed the same order.

Although discrimination against ^15^N by NR is thought to exceed discrimination by GS (Hu and Guy, 2020), we observed greater overall discrimination in the ammonium experiment than in the nitrate experiments. Higher whole-plant discrimination indicates greater efflux of unassimilated N back to the rooting medium – in other words, a greater futile cycling of inorganic nitrogen across root cell membranes. Our IMB model analysis indicates that the average root *E*/*I* was 0.09 in the low nitrate experiment, 0.31 in high nitrate experiment and 0.41 in the ammonium experiment. A much greater futile cycling for ammonium as compared to nitrate has been commonly observed in many plants (Britto and Kronzucker, 2001), and, at higher concentrations, may contribute to ammonium toxicity (Britto et al., 2001). A recent study from Hachiya et al. (2021), however, has attributed ammonium toxicity in *Arabidopsis* to acidification caused by overly high rates of $NH_{4}^{+}$ assimilation catalyzed by a plastidic GS.

Measuring *E*/*I* has been difficult because of high spatial and temporal heterogeneity in actively growing plants (Hawkins and Robbins, 2010). Due to the dynamic nature of N flux in time and space within the plant, an integrated approach to assess *E*/*I* would be useful. There are two available methods that can measure *E*/*I* directly, namely with microelectrodes (Hawkins and Robbins, 2010) or by the CATE method using either ^13^N or ^15^N as a tracer (Kronzucker et al., 1995; Min et al., 1999; Kalcsits and Guy, 2016a). These methods provide instantaneous measures of nitrogen flux, whereas the IMB model is expected to better reflect the time-integrated (and therefore potentially less variable) efficiency of nitrogen uptake. Nonetheless, Kalcsits & Guy (2016) compared *E*/*I* of balsam poplar as measured by the CATE method and by the IMB model under 0.5 mM ammonium or nitrate, and in both cases found that *E*/*I* was higher when plants were provisioned with ammonium than with nitrate. *E*/*I* is also known to increase as substrate concentration increases, as suggested by our nitrate experiments. Min et al. (1999) measured *E*/*I* at 0.1 mM and 1.5 mM nitrate in trembling aspen (*Populus tremuloides* Michx.), lodgepole pine (*Pinus contorta* Dougl.) and Douglas-fir (*Pseudotsuga menziesii* (Mirb.) Franco), and in every case found *E*/*I* was increased at the higher concentration.

An advantage of the IMB model over other methods to measure nitrogen flux is the information it provides on relative concentrations of inorganic and organic nitrogen translocated in the xylem and the proportioning of N assimilation between roots and shoots. This information is calculated from the leaf-root differences in discrimination (Equation 4) and by combining it with the organ level data on N partitioning (Equation 7). In the nitrate experiments, leaf Δ^15^N values, on average, were both close to zero, while stem Δ^15^N and root Δ^15^N were not. We found similar patterns in nitrate-grown black cottonwood and heart-leaved willow (Hu and Guy, 2020; Hu et al., 2022). Leaf Δ^15^N in these circumstances is close to zero because the transported ^15^N-enriched inorganic N and ^15^N-depleted organic N from roots essentially balance each other out. In the ammonium experiment, however, leaf Δ^15^N was 6.0‰ and there was a much smaller leaf-root difference in Δ^15^N. This smaller difference is expected since, in most species, ammonium is primarily assimilated in roots and not leaves, whereas the site of nitrate assimilation is more variable (e.g., Kalcsits et al., 2015). The overall mean *Ti*/*Tt* in the ammonium experiment was 0.07, and *P_root_* was 0.97. In contrast, in the low nitrate experiment *Ti*/*Tt* and *P_root_*, averaged 0.23 and 0.82, and in the high nitrate experiment, they averaged 0.42 and 0.71, respectively.

Another profound advantage of the IMB model is its utility for screening large numbers of plants. The assessment of genotypic variation in *E*/*I* and other flux parameters using the CATE or microelectrode methods has been limited to a few genotypes (e.g., Abdellaoui et al., 2001) or to species with known contrasting NUE (Britto and Kronzucker, 2001; Britto et al., 2001; Glass, 2003). In the present study, we used the IMB model to assess 23 historical canola lines. Although we were unable to detect differences between specific lines because of low statistical power (there were only 3-4 individuals tested per line), our hypothesis that direct selection for yield in canola may have resulted in the indirect selection on N-uptake efficiency (i.e., 1- *E*/*I*) was supported by the group differences we found in the ammonium experiment. In this experiment, the CH group had better growth than the OP group, as well as higher Δ^15^N at the root, stem, leaf and whole-plant levels. The commercial hybrids also had a greater mean leaf-root Δ^15^N difference. These differences indicate that the commercial hybrids, which are more recent than the open-pollinated lines, tend to have higher *E*/*I* and *Ti*/*Tt*, and a lower *P_root_*. The higher *Ti*/*Tt* and lower *P_root_* suggest that hybrid lines have a slightly greater capacity for ammonium translocation in the xylem and proportionally greater ammonium assimilation in the leaves. The greater relative growth of these lines when provisioned solely with ammonium also suggests increased resistance to ammonium toxicity. In contrast, the slightly higher *E*/*I* suggests more futile cycling at the soil-root interface and a modest reduction in uptake efficiency. This result is surprising, as we might initially expect a greater growth demand to result in relatively less N leakage, not more. Overall, the slightly higher efflux implies an even faster rate of ammonium uptake by the CH group. By harvest, we calculate that gross N influx and efflux averaged 244±18 and 97±14 mg N per plant for the OP group, and 430±38 and 182±33 mg N per plant for the CH group, respectively.

The greater stem Δ^15^N relative to root Δ^15^N observed in the ammonium experiment underscores an important limitation of the current IMB model in assuming that the root inorganic N content is negligible. Kalcsits et al. (2014) noted that this assumption must result in an underestimation of the isotopic difference between root- and leaf-assimilated organic N, and thus an underestimation of *Ti*/*Tt* with consequent effects on the estimation of *P_root_* and, to a lesser extent, *E*/*I*. They also noted that the fraction of inorganic N in roots typically varies from 1 to 10% of the total N (Evans et al., 1996; Yoneyama et al., 2001; Black et al., 2002; Kolb and Evans, 2003). The root inorganic and organic N fractions are expected to differ in Δ^15^N by an amount that approximates the discrimination factor of the assimilatory enzyme. Accordingly, Hu and Guy (2020) found that the Δ^15^N of the soluble organic and inorganic N fractions in roots of nitrate-grown black cottonwood differed by 18.2‰. Given that the Δ*_enzyme_* for GS is 17‰, and if as much as 1/10^th^ of the root N content is ammonium, the whole root discrimination could be decreased by up to 1.7‰. Indeed, if N assimilation were fully restricted to the roots, the aerial portions of the plant would show greater discrimination than the roots by an equal amount. If only stems show greater discrimination, as in our ammonium experiment (**Table 2**), then some N assimilation must also occur in the leaves but with little of it making its way into other tissues. We note from **Table 2** that the average leaf and stem Δ^15^N values for the OP and CH groups were very similar and did not differ, whereas the root Δ^15^N for the CH group was significantly greater than it was for the OP group. The difference in root Δ^15^N may therefore indicate that the CH roots have a lower load of unassimilated ammonium than the OP roots and, if accounted for, could reduce or even eliminate the differences in *E*/*I*, *Ti*/*Tt* and *P_root_*. Direct measurements of root ammonium contents are needed to evaluate this possibility.

Contrary to our hypothesis, there were no group differences in C/N ratio (our proxy for NUE) or in whole-plant δ^13^C (our proxy for WUE). We estimated the genetic correlations between key physiological traits and biomass across all 23 canola lines. The trait-to-trait correlations varied considerably between experiments (**Figure 3**). There appeared to be a trade-off between NUE and WUE in the low nitrate experiment, but the proxies for these traits were positively correlated in the ammonium and high nitrate experiments. Consistent with the group differences noted above for the ammonium experiment, whole-plant biomass was positively correlated with *Ti*/*Tt* and negatively with *P_root_*. The root:shoot ratio was not correlated to any N-related traits in the ammonium and low nitrate experiments, but it was positively correlated with *P_root_* and *E*/*I* in the high nitrate experiment. The correlation with *P_root_* might be expected mathematically (i.e., increased root assimilation simply because of root biomass), but the calculation of *E*/*I* is independent of root mass. Increased root-to-shoot ratio was also correlated with increased *E*/*I* in heart-leaved willow (Hu et al., 2022) and in black cottonwood (Hu, 2022). A higher root biomass might result in a higher N supply (greater uptake) relative to N demand by the shoot (e.g., for photosynthetic proteins), allowing more unassimilated nitrate to efflux from the root (Evans, 2001).

## Conclusion

This paper represents the first use of the IMB model to assess time-integrated nitrogen-use traits in a commercial crop. Relative to older open-pollinated lines, modern canola hybrids appear to be collectively able to better utilize ammonium as their sole nitrogen source. Selection for yield in canola may have resulted in this change. In western Canada, urea is the predominant source of nitrogen used to fertilize canola (Mahli et al., 2007). Given that urea is converted to ammonium by urease enzymes and hydration in the soil (Bremner and Krogmeier, 1989), intensive mono-cropping may have favored an increased ability to tolerate and/or utilize ammonium.

## Data availability statement

The raw data supporting the conclusions of this article will be made available by the authors, without undue reservation.

## Author contributions

YH performed hydroponics experiments, analyzed data, and drafted the manuscript. RDG provided scientific insight into data analysis and edited the manuscript. RYS conceived of the study, obtained funding, and edited the manuscript. All authors contributed to the article and approved the submitted version.

## Funding

This work was funded by the Canola Agri-Science Cluster (2018-23), a partnership between Agriculture and Agri-Food Canada (AAFC) and the canola industry (SaskCanola, Alberta Canola, Manitoba Canola Growers and Canola Council of Canada) under the Canadian Agricultural Partnership to RYS. Research for RDG was funded by the Natural Sciences and Engineering Research Council of Canada (NSERC) Discovery grant GR004049. YH received stipends through the Research Affiliate Program of AAFC.

## Acknowledgments

We would like to thank Sally Vail (*B. napus* breeder, AAFC-Saskatoon) and the canola industry partners for providing the seed material, Brett Hill (AAFC-Lethbridge) for assistance with stable isotope analysis, and Melina Biron (UBC-Vancouver) for advice on hydroponic system design.

## Conflict of interest

The authors declare that the research was conducted in the absence of any commercial or financial relationships that could be construed as a potential conflict of interest.

## Publisher’s note

All claims expressed in this article are solely those of the authors and do not necessarily represent those of their affiliated organizations, or those of the publisher, the editors and the reviewers. Any product that may be evaluated in this article, or claim that may be made by its manufacturer, is not guaranteed or endorsed by the publisher.
